# Online exams and the COVID-19 pandemic: a hybrid modified FMEA, QFD, and *k*-means approach to enhance fairness

**DOI:** 10.1007/s42452-021-04805-z

**Published:** 2021-09-28

**Authors:** Hamid Haghshenas Gorgani, Sharif Shabani

**Affiliations:** 1 Engineering Graphics Center, Sharif University of Technology, Tehran, Iran; 2 Mechanical Engineering Department, Sharif University of Technology, Tehran, Iran

**Keywords:** COVID-19, Online exam, Academic stress, Higher education, Fairness

## Abstract

COVID-19 pandemic caused an increasing demand for online academic classes, which led to the demand for effective online exams with regards to limitations on time and resources. Consequently, holding online exams with sufficient reliability and effectiveness became one of the most critical and challenging subjects in higher education. Therefore, it is essential to have a preventive algorithm to allocate time and financial resources effectively. In the present study, a fair test with sufficient validity is first defined, and then by analogy with an engineering product, the design process is implemented on it. For this purpose, a hybrid method based on FMEA, which is a preventive method to identify potential failure modes and prioritize their risk, is employed. The method's output is provided to the QFD algorithm as the needs of product customers. Then, the proposed solutions to prevent failures are weighted and prioritized as the product's technical features. Some modifications are made to the classic form of FMEA in the proposed method to eliminate its deficiencies and contradictions. Therefore, our proposed algorithm is a precautionary approach that works to prevent breakdowns instead of fixing them following their occurrence. This issue is very effective in increasing the efficiency of activities in times of crisis. Eventually, a prioritized list of preventive actions is provided, allowing us to choose from available solutions in the circumstances with limited time and budgetary, where we cannot take all possible actions.

## Introduction

The COVID-19 pandemic, also known as the Coronavirus pandemic, is an ongoing global crisis that caused significant alterations to academia, demanding new regulations and creating unprecedented challenges for both learners and tutors [1]. In order to minimize the transmission of the contagious virus, students have to study from home. Education systems need to provide online system strategies for teaching, learning, and evaluation methods to help with this transition. Besides the current demand for online education as an effect of the pandemic, some of the new practices imposed by the current pandemic situation can be maintained and used even after the crisis [2]. Investigation and analysis of how pandemic effects academic activities help us overcome current challenges. We can use this experience to enhance our academic measures and advance online education capabilities [3].

With the outbreak of Coronavirus (COVID-19) disease, online exams became common practice for academic evaluation. Online exams offer several desirable advantages such as time efficiency [4], ease of use [5], enhanced adaptability [6, 7], and provision of immediate feedback [8]. On the flip side, computer and internet accessibility [9], lack of experience with computer or online assessment processes [10], test anxiety [11], and higher cheating rates [12, 13] are some of the main challenges that come with online exams.

Given the critical pandemic situation, online exams are inevitable and will increase even in non-critical situations. Therefore, in order to hold them more fairly, methods should be considered, and possible failures should be identified to be mitigated or eliminated as a precaution. Therefore, the basic questions, or in other words, the objectives of our research, are as follows:

- What is the definition of a fair exam?
- Who are the customers of an online test process, and what are their needs?
- What is the priority and importance of each of these needs for them?
- What characteristics of the process can be effective in meeting these needs, and to what extent?

The ultimate goal is to provide a list of things that we can do to have a fairer online exam.

Fairness is often regarded as the most important pillar of examinations, which strongly affects students [14, 15]. Exam fairness preserves academic integrity and improves the students' motivation to enhance their performance [16, 17]. There are numerous challenges to fairness in online exams, such as limited proctoring options and higher cheating rates [18].

The current circumstances and the necessity to employ online exams while eliminating their shortcomings exhibit the demand for an effective algorithm. Failure mode and effects analysis (FMEA) can be a robust tool for this matter. FMEA is a widely used technique to diagnose and prevent product, system, and operation failure modes before occurrence [19]. As Lolli et al. mentioned in their work in 2016, FMEA is primarily performed by providing a list of potential failure modes, assigning numbers associated with the severity, detection, and probability of occurrence to each of these events, and eventually obtaining the risk priority number or RPN from the multiplication of these numbers. The performance of FMEA relies entirely on proper determination of the numbers of intensity, detection, and occurrence, and thus the RPN values. For the intensity and detection numbers, which are essentially subjective values, this is less of a challenge than the occurrence number, which has an objective nature [20].

K-means clustering method is one of the plainest yet most commonly used unsupervised intelligent learning algorithms. It can help us prevent conflicting situations, especially in the assignment of occurrence probability numbers [20].

In 2002, Berget and Naes [21] introduced a fuzzy K-means-based clustering algorithm for sorting raw materials to improve the quality of the final product, which works similarly to an optimization problem. In 2004, Sarkar [22] proposed a clustering algorithm for failure modes to investigate the probabilities of each state occurring*.* Also, in 2014, Lolli et al. [23] presented an application of K-Means for sorting according to multi-criteria classification, the key information of which can be the basis for presenting an algorithm with our intended purpose. In a similar work in 2016, Lolli et al. [20] used K-Means to resolve inconsistencies in the "occurrence" parameter, which is a subjective parameter of FMEA.

In the last step of the FMEA method, a list of preventive and corrective actions is presented to mitigate the occurrence, minimize the effects, or enhance the probability of detecting improper conditions [24]. Contrarily, time and cost limitations make it impracticable to use all offered solutions to eliminate every unfavorable situation. Therefore, we need to rank and prioritize recommended corrective actions.

Quality function deployment (QFD) is an effective and robust means commonly used to design engineering products aiming to reach maximum customer satisfaction. In this method, customer needs are associated with the product's technical characteristics in the QFD matrix. Eventually, the QFD process results in a ranked and weighted list of technical product features [25, 26]. In an analogy, failure modes are regarded as customer needs, the RPN as the priority of needs, and the listed preventive and corrective actions as the product features. These entities are supplied to the QFD. The final goal of this algorithm is to present a weighted list of corrective and preventive actions as the output.

In the event of crises such as the Covid-19 pandemic and the increasing demand for online testing, along with time and resource limitations that are more severe at this time, it is essential to have a preventive algorithm for the effective allocation of financial and time resources. The main innovation of the proposed algorithm is to simulate the online test process with an engineering product and then simultaneously use tools FMEA, K-Means, and QFD to design it. The most important advantages of such an algorithm are as follows:

The proposed method in this research first identifies all groups that are internal or external customers of this process. It is based on a survey of all customers, to be a comprehensive approach. One of the basic foundations of the proposed method is FMEA, which is inherently preventive in nature. Therefore, our algorithm is preventive and so deals with the prevention of the faults, instead of repairing them after their occurrence. This issue is very effective in increasing the efficiency of activities in times of crisis. Also, FMEA has contradictions that have been largely resolved in the proposed algorithm using K-Means. Employing QFD, as a tool based on maximum customer satisfaction, is very efficient in resource allocation. Therefore, time and financial resources, that are limited especially in times of crisis, will be spent on activities that ultimately lead to greater process customer satisfaction.

The proposed algorithm has been implemented on mechanical engineering students at the Sharif University of Technology for two consecutive semesters. This paper aims to improve exam fairness by analyzing the worries and challenges that students of the Sharif University of Technology have experienced during their online exams in times of the COVID-19 pandemic. The results are presented and investigated in this paper.

## Materials and methods

We aim to provide an algorithm that can be deployed to identify existing and potential defects of a fair online exam. Then, find and prioritize possible solutions. The prioritization is necessary since it is impossible to implement all possible solutions regarding time and cost limitations. So, we can only apply the most effective solutions and disregard less effective ones.

For this, it is necessary to define a fair exam at first, and then, according to its characteristics, potential failure modes and their effects should be identified. Solutions to eliminate or reduce the effects should be provided and prioritized.

### Definition of a fair exam

In an online survey, we asked college students and professors to provide their definitions of a fair exam. Additionally, they were requested to list potential problems that they have encountered, describe their effects, and suggest solutions for more fairness. Twelve university professors and 118 students participated in the survey. In order to have a relatively homogeneous statistical population that covers a broad spectrum, in the group of professors, three people are in mathematics and engineering, three in medicine, three in humanities, and three in art. Three people in each category included a highly experienced professor (more than 20 years of experience), a moderate professor (between 10 and 20 years of teaching), and a young professor (less than 10 years of experience). Also, from each of the disciplines mentioned in the professors' group, 30 students were selected with a combination of 10 students with a GPA of A, 10 students with a GPA of B, and 10 students with a GPA of C. In the art group, the survey of two students with a GPA of C was invalid and resulted in a total of 118 students. Summarizing the commonalities and rewriting their views led to the following definition:

A fair assessment occurs when participants' knowledge of the presented topics is measured appropriately, they have equal conditions, and they are fully justified with the outcome [15]. Moving toward the above expressions will lead to a fairer exam.

### Basic FMEA

FMEA is a powerful engineering tool for the identification of potential failure modes and their sources. This process is done through thinking about a product, process, or service in reverse [27]. In this method, the effect of each failure mode on the customer is represented by the severity number (*S*). Likewise, the likelihood of detecting a failure when it occurs is shown by the detection number (*D*), and the probability of its occurrence is reported by the occurrence number (*O*). These three numbers lie within the range of 1 to 10. Higher severity and probability of occurrence lead to larger *O* and *S* numbers. The *D* number becomes larger when preventive detection of the failure mode is unlikely. The risk priority number (RPN) is:

1 $${\text{RPN}} = S \times D \times O$$

where RPN ranges between 1 and 1000, and higher numbers indicate a risk of the failure mode [28–31]. The scales used to determine the *S*, *O*, and *D* values are provided in Table 1 [32, 33].

**Table 1:** FMEA Scale for severity (S), occurrence (O), and detection (D) numbers [32, 33]

Probability of occurrence	Rating	Severity (S)	Rating	Detectability (D)	Rating
Almost never	1	No	1	Almost certain	1
Remote	2	Very slight	2	Very high	2
Very slight	3	Slight	3	High	3
Slight	4	Minor	4	Moderately high	4
Low	5	Moderate	5	Medium	5
Medium	6	Significant	6	Low	6
Moderately high	7	Major	7	Slight	7
High	8	Extreme	8	Very slight	8
Very high	9	Serious	9	Remote	9
Almost certain	10	Hazardous	10	Almost impossible	10

### Modifying basic FMEA using K-means clustering

**Fig. 1:**
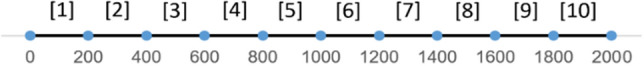
Occurrence classes in the mentioned example (with a minimum of 0 and a maximum of 2000 occurrences per year)

Proper determination of RPN relies on the correct assignment of *S*, *D*, and *O* values. The nature of these numbers implies that *S* and *D* are subjective, but *O* is objective. Particularly, the magnitude of *O* depends on the occurrence records of a failure mode. Suppose that *O* values lie within the range of 1–10, which suggests that occurrence probabilities are divided into ten distinctive classes. Consequently, if a type of failure occurs up to 2000 times a year, the range of each class will be 200. This is shown in Fig. 1.

Now, assume a failure mode occurs 596 times, and another failure mode happens 604 times. In this case, the first failure will be in the third class, while the second one will be in the fourth class, knowing that it happened only eight times more than the first one. This paradox casts doubt on the accuracy of occurrence number assignments.

We use the intelligent, nonlinear clustering method of *k*-means to resolve this issue. In this algorithm, *k* cluster centers are randomly selected, where *k* is user-specified. In the next step, the Euclidean distance between each point and the cluster centers is measured. Each point is assigned to the cluster with the most adjacent center. When all existing points are allocated, new centroids are recalculated by averaging between each cluster's members. When all existing points got allocated to different centers, new centers are recalculated by averaging between each cluster's members. This process continues until the predetermined ending condition is fulfilled [34]. K-means clustering method is an unsupervised learning algorithm [35, 36]. For assigning the Occurrence number, we divide the range into ten classes. In order to assign the Occurrence number, the range was divided into ten classes. Then, the midpoint of each class, along with other data points, was given to *k*-means as input. Since *k*-means does not leave any cluster empty, this process excludes the risk of placing two points with a close number of occurrences in two separate clusters. Similarly, it is unlikely for two far values to end up in two consecutive classes. Consequently, the paradox with the results will be resolved [37].

### Modifying risk priority numbers using fuzzy logic

High intensity, regardless of RPN, means high risk [38]. Because, even if the probability of its occurrence is low or the possibility of its preventive detection is high, it can lead to adverse effects on the process customers. Therefore, risky situations are the sum of failure modes with a high RPN plus high severity cases. The combination of these two factors can be done in different methods, but it depends entirely on the nature of the factors and the way of human inference. In such conditions, the closest tool to human inference is a fuzzy logic-based system [39].

The most common concepts of fuzzy systems are pure fuzzy, fuzzy Sugeno Takagi base, and Mamdani base [33, 40]. In the case of human inferences, which require the use of expert knowledge with linguistic variables, fuzzification of them, inference, and then defuzzification, the most appropriate option is fuzzy systems based on the Mamdani algorithm [15].

**Fig. 2:**
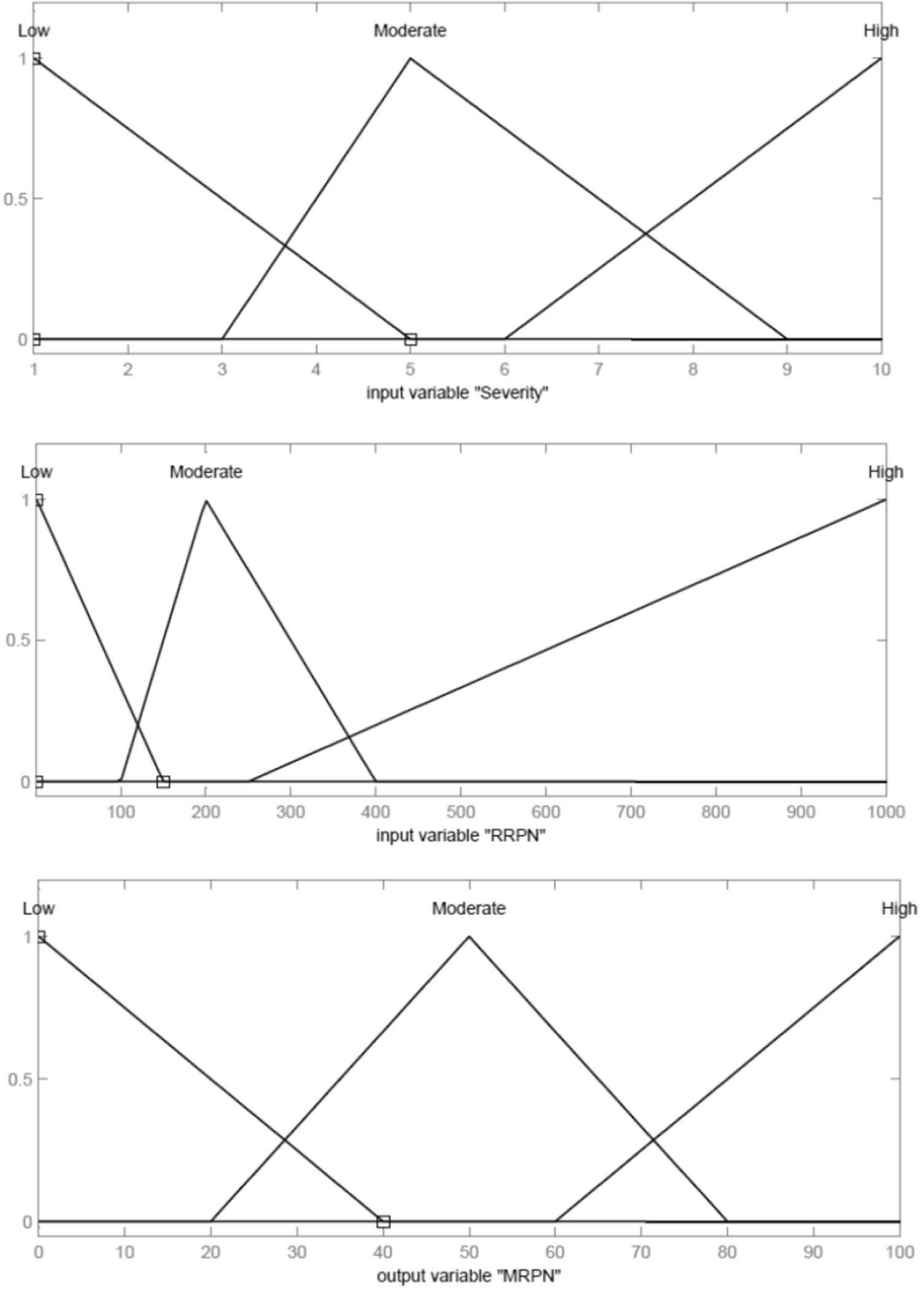
Shape of the membership functions of **a** input variable “Severity”, **b** input variable “RRPN”, and **c** output variable “MRPN” for fuzzy system “Risk”

To achieve this goal, a fuzzy inference system has been formed, with two inputs and one output. The shape of the membership functions of the inputs and output, which are of type Trimf (Triangular-shaped membership function), is as shown in Fig. 2.

Also, the fuzzy rules and its inference system are as follows:

1. *If (Severity is Low) and (RRPN is Low) then (MRPN is Low).*
2. *If (Severity is Low) and (RRPN is Moderate) then (MRPN is Low).*
3. *If (Severity is Low) and (RRPN is High) then (MRPN is Moderate).*
4. *If (Severity is Moderate) and (RRPN is Low) then (MRPN is Low).*
5. *If (Severity is Moderate) and (RRPN is Moderate) then (MRPN is Moderate).*
6. *If (Severity is Moderate) and (RRPN is High) then (MRPN is High).*
7. *If (Severity is High) then (MRPN is High).*

**Fig. 3:**
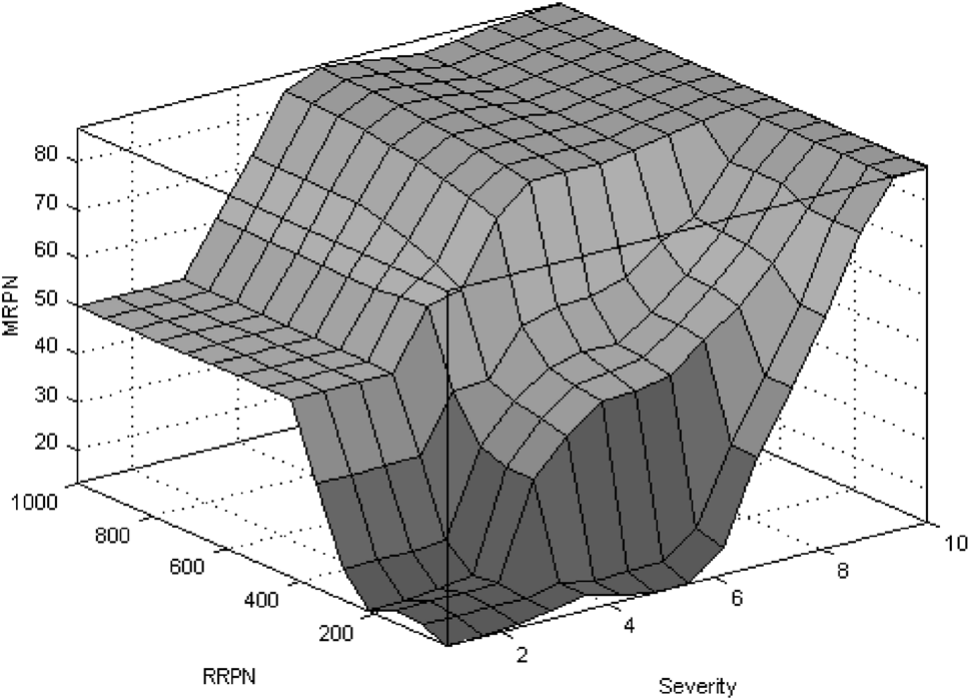
Surface plot for fuzzy system “Risk”

The result of fuzzy rules and the relationship of the inputs to the output is according to the surface drawn in Fig. 3.

Therefore, if we call this fuzzy system as "Risk", we can say that:

2 $${\text{RRPN}}_{j} = {\text{Risk}}\left( {{\text{MRPN}}_{j} \cdot S_{j} } \right)$$

where MRPN is the modified value of RPN, assuming the high values of severity are risky, and MRPN is in the range of 0 and 100.

### Prioritizing actions using QFD

After determining the RPN value, possible preventive and corrective actions are determined for each failure mode. The quality function deployment (QFD) is used to determine the priority of each proposed solution. QFD is a customer-oriented method in designing new engineering products, aiming to maximize customer satisfaction [26, 27, 41]. The main idea of QFD is to provide a list of prioritized customer needs related to the product. Then, the technical characteristics of the product are specified. The QFD matrix, shown in (3), is the mapping of needs to technical characteristics of the product [25, 42, 43]:3where *W*_*ij*_ shows how much the *j*th technical characteristic meets the *i*th need. *A*_*j*_ is a technical characteristic, and *R*_*i*_ is the priority number for *i*th need. Now, the weight of each technical feature is calculated by Eq. (4):

4 $$W_{j} = \mathop \sum \limits_{j = 1}^{m} R_{i} W_{ij}$$

5 $$W_{j}^{N} = \frac{{W_{j} }}{{\mathop \sum \nolimits_{j = 1}^{n} W_{j} }} = \frac{{\mathop \sum \nolimits_{i = 1}^{m} R_{i} W_{ij} }}{{\mathop \sum \nolimits_{j = 1}^{n} \mathop \sum \nolimits_{i = 1}^{m} R_{i} W_{ij} }}$$

Equation (5) shows the normalized weight.

### The proposed algorithm

In an analogy with the design of an engineering product, the steps for performing the proposed algorithm will be as follows:

*Step 1* Identifying the Customers:

Customers of an online exam process fall into two categories: "professors and assistants" as group *A* and "students" as group *B*. In expressing the reason for classification and in an analogy with engineering products, process customers can be classified into two categories: "Manufacturers and Service Providers" (domestic customers) and "Consumers" (foreign customers). Here, professors and assistants are as manufacturers and service providers and students as consumers. Also, their opinions about possible failure modes are considered as the voice of the customer (VOC) or customer complaint. Suppose the number of people in group *A* is *N*_*A*_, and the number of people in group *B* is *N*_*B*_.

*Step 2* Exploration of potential failure modes:

Using a survey of groups *A* and *B*, all possible failure modes are identified. Each failure mode is called *F*_*j*_. Suppose the total number of failure modes is *m*. Therefore:

6 $$F = \left[ {F_{j} } \right],\quad j = 1\;{\text{to}}\;m$$

where *F* is the set of failure modes.

*Step 3* Determine severity numbers (S):

For each *F*_*j*_, determine the values $\overline{S}_{A}^{j}$ and $\overline{S}_{B}^{j}$, which are the average severity assigned to that failure mode by the individuals in groups *A* and *B*, respectively. Then calculate the value of *S*_*j*_ according to Eq. (7):

7 $$S_{j} = \frac{{\overline{S}_{A}^{j} + \overline{S}_{B}^{j} }}{2}$$

*Step 4* Determine detection numbers (D):

For each *F*_*j*_, the *D*_*j*_ value is determined, which is the average of the detection number assigned to that failure mode by individuals in group *A*. (In this case, the poll is conducted only from group *A*).

*Step 5* Identify the repetition of each failure mode:

$q_{j}^{A}$ Is the value which the failure mode *F*_*j*_ is repeated in group *A*, and $q_{j}^{B}$ is the same value in group *B*. *q*_*j*_, number of repetitions of failure mode *F*_*j*_, is calculated from Eq. (8):

8 $$q_{j} = q_{j}^{A} + q_{j}^{B}$$

*Step 6* Calculate the central points of the occurrence intervals:

The maximum and minimum values of *q*_*j*_ obtained in step 5 are called *q*_max_ and *q*_min,_ consequently. Therefore, the center of each occurrence interval can be calculated from (9):

9 $$q^{\prime }_{l} = q_{\min } + \left( {2l - 1} \right)\left( {\frac{{q_{\max } - q_{\min } }}{20}} \right)$$

where $q^{\prime }_{l}$ is the center of the *l*th interval, and l is a digit from 1 to 10.

*Step 7 *Calculating Occurrence values (O), using k-means:

Assume the set *Q* as Eq. (10):

10 $$Q = \left\{ {q_{j} \cdot q^{\prime } } \right\}, \quad l = 1\;{\text{to}}\;10,\quad j = 1\;{\text{to}}\;m$$

Then using Matlab-R2013 software, *O*_*j*_ = *k-means (Q, 10)*, obtain the results where, *O*_*j*_ is the number of cluster and shows the occurrence value.

*Step 8* Calculate raw RPN (RRPN) value for each failure mode:

Using Eq. (1), ${\text{RRPN}}_{j}$ values for each *F*_*j*_ are calculated (${\text{RRPN}}_{j} = S_{j} \times D_{j} \times O_{j}$).

*Step 9* Determine Modified RPN (MRPN) value using fuzzy inference system:

Determine MRPN using Eq. (2) by applying fuzzy inference system “Risk”.

*Step 10* Extract the possible solutions of each Fj:

This is done using a survey of people in both groups *A* and *B*. Similar and close values are conceptually unified. *R* indicates the total number the solutions (preventive and corrective actions), which we present as the set *C*:

11 $$C = \left[ {C_{r} } \right] , \quad r = 1\;{\text{to}}\;R$$

where *C*_*r*_ is the *r*th solution.

*Step 11* Forming a QFD matrix:

In an analogy to the engineering products, failure modes of online academic exams are given as the customer needs. Here, priority number of each customer demand is MRPN of each failure mode (MRPN_*j*_), and suggested solutions will be the product technical characteristics (*C*_*r*_). To fill the matrix, we acquire the average values from groups *A* and *B*. Therefore:12where *W*_*jr*_ is the effect of the solution *C*_*r*_ on the failure mode *F*_*j*_. According to (3), the weight of each solution (*W*_*r*_) will be as (13):

13 $$W_{r} = \mathop \sum \limits_{j = 1}^{m} \left( {{\text{MRPN}}_{j} \times W_{jr} } \right)$$

And, the normal weight of each solution ($W_{r}^{N}$) is:

14 $$W_{r}^{N} = \frac{{W_{r} }}{{\mathop \sum \nolimits_{r = 1}^{R} W_{r} }} = \frac{{\mathop \sum \nolimits_{j = 1}^{m} \left( {{\text{MRPN}}_{j} \times W_{jr} } \right)}}{{\mathop \sum \nolimits_{r = 1}^{R} \mathop \sum \nolimits_{j = 1}^{m} \left( {{\text{MRPN}}_{j} \times W_{jr} } \right)}}$$

*Step 12* Prepare a list of preventive and corrective actions along with their priorities:

A prioritized list containing the set $C = \left\{ {C_{r} } \right\}$, is presented as the result of the algorithm. $W_{r}^{N}$ shows the solution's weight, which also indicates its priority.

## Results

Before implementing the proposed algorithm, as mentioned in Sect. 2.1, a survey was conducted to define a fair exam. At the same time, the most significant aspects of the impairment of this definition were asked, and the following 12 attributions were derived:

- The questions are fully related to the topics
- The duration of the exam is reasonable
- Cheating is prevented
- Appropriate references are taken for evaluation
- Students have equal access to hardware and software facilities
- Questions' demands are clear
- If the questions vary for each student, the level of difficulty should be the same for all of them
- Scores are distributed reasonably
- The results are justifiable
- A Clear statement of evaluation policies and exam details is given before the test
- The level of questions is proportional to the level of teaching
- Appropriate time and location are considered for the test.

It is worthy to note that another customer of the process is the "educational system", whose needs are hidden within the needs of the two mentioned groups, with the aim of not prolonging the content and diverging the results. For example, we can say the relevance of the exam content to the taught topics and appropriate references ensures that the training is in line with the objectives of the education system. Prevention of widespread cheating in the exam guarantees the validity of the training provided by the educational system, and clarifying the demands for exams follows the goals of the education system.

Then, the proposed algorithm was implemented in two consecutive semesters (spring 2020 and fall 2021). 80 people, including 60 students, 8 professors, and 12 teaching assistants (20 people in group A and 60 people in group B), participated in it. Based on steps 1 and 2, the results show that a total of 33 potential failure modes (Matrix F) are given in Table 2 (column 3).

**Table 2:** Potential failure modes and their causes

Row	Attributions	Failure modes	Causes
1	The questions are from topics related to the lesson	F1: Questions are from marginal topics	Questions are not designed by the educator
			The educator believes marginal questions can prevent cheating
			The professor wants to assess students' attention in the class
			Class discussions are marginal
		F2: Questions are from untaught topics	Questions are not designed by the educator
			The educator has too much expectations from students
			The educator is not teaching according to the syllabus
			The educator is not in coordination with other groups
			The teacher teaches different subjects in different semesters
		F3: Questions are not distributed over the topics	Questions are not designed by the educator
			To avoid cheating, the teacher asks questions from the sections that are most challenging
			Lesson is taught by several instructors but not all of them are present in the evaluation design
			The teacher is more interested in some particular topics
			More questions can be asked from a topic
			The teacher cannot convey the subject well
2	The duration of the exam is reasonable	F4: Short exam time	The educator reduces the exam time to prevent cheating
			The exam designer does not have an accurate assessment of the amount of time required to solve the questions
			Questions are too hard
			Lots of calculations are needed
			the instructor has reduced the exam time to sort and distinguish the students
		F5: Not providing enough time for uploading the answers	To prevent cheating, the teacher will greatly reduce the time required to submit responses
			Low quality internet connection
			The educator does not now the time required for uploading the answers
			Uploading system is not functioning well
			Some students may not have good quality facilities
		F6: Technical problems of the examination system reduce the efficient time of the test	Students cannot go back to previous questions during the exam
			Bad system design
			System is being overused
			Haste in developing examination systems
		F7: Too much time for the test	Not checking the required time for the exam
			considering too much time to submit answers
3	Cheating is prevented	F8: Using unauthorized online sources	Memorization questions in place of conceptual questions
			There is no proctoring
			Student's lack of moral commitment
		F9: Using unauthorized text books	Student's lack of moral commitment
			Memorization questions in place of conceptual questions
		F10: unauthorized consultation	Student's lack of moral commitment
			There is no proctoring
		F11: Having someone else to take the test	Student's lack of moral commitment
			No way to authenticate the examiner
		F12: Using unauthorized accessories	Student's lack of moral commitment
			improper question design
		F13: Sending the answers after the deadline	Student's lack of moral commitment
			The educator is taking things too easy
4	Appropriate reference for evaluation	F14: inconsistency in grading	Prior knowledge of the corrector may affect the correction process
			Different mental conditions of the grader
			Too easy/hard questions
		F15: Wrong reference for grading	The test key is not written by the test designer
			The educator makes a mistake in solving the questions
5	Equal hardware and software facilities	F16: Unequal hardware facilities among students	Inability of some students to provide appropriate facilities
		F17: Low quality internet connection	Student's inability to provide a strong internet connection
			Improper infrastructure
6	Clarity of evaluation requirements	F18: Vague questions	Improper design of questions
			Exam design in a different language from the teaching language
		F19: Ambiguity of question correction criteria	Lack of clear explanations by the teacher
			The educator fails to decide criterions
7	Same difficulty level	F20: The method used to reproduce the questions changes the difficulty level of the questions	Inappropriate methods for reproducing questions
8	Proportional distribution of results	F21: The range of scores is discrete and has a gap (deviation from the standard is high)	Teaching is not well done
			Students have distinctly varying knowledge levels
			System problems affecting the grade
		F22: The average score is too low	The test is inappropriate and has unreasonable difficulty
			Students are not well prepared for the exam
			Insufficient time
			System and internet problems
			Teaching is not well done
		F23: The average score is too high	The test is inappropriate and has unreasonable difficulty
			Cheating has happened
		F24: Marks are too close together and not evenly distributed	Students have a similar level
			Grading steps are not small enough
			Questions are not appropriate and cannot distinguish the students
9	The results are justifiable	F25: Students do not accept assessment policies	Insufficient explanations of the educator
			Improper explanations of the educator
			The educator does not provide correct answers to students after the test
			The professor does not give students a chance to complain
		F26: Student objections cannot be responded	The irrationality of the educator
			Educator's misconception of his own performance
10	Clarification of evaluation policies before the test	F27: Ambiguity of evaluation policies	Insufficient explanations of the educator
			Improper explanations of the educator
		F28: Lack of clarity or change in the layout and importance of the taught topics	Insufficient explanations of the educator
			Improper explanations of the educator
		F29: Uncertainty of test duration	Insufficient explanations of the educator
			Improper explanations of the educator
11	The level of questions is proportional to the level of teaching	F30: Questions are more difficult than usual comparing to taught topics	The educator wants to prevent cheating by making questions hard
			The educator wants to show off
		F31: Questions are easier than usual comparing to taught topics	Inadequate teacher's understanding of class preparation
			The topics taught were very difficult, the exam was routine
12	Appropriate time and place conditions	F32: The exam is held at times when students are typically less mentally prepared	Lack of proper planning of college education for exam hours and dates
			Educator's lack of sympathy and mutual understanding
		F33: Stressful environment	Insufficient time
			Educator's inappropriate behaviors
			Too hard questions
			Improper presentation of questions
			The educator is not present at the exam session

For more clarifying, these failure modes are classified into these 12 attributes (Column 2). Also, the causes for each one (obtained through surveys) are given in the fourth column of this table.

**Fig. 4:**
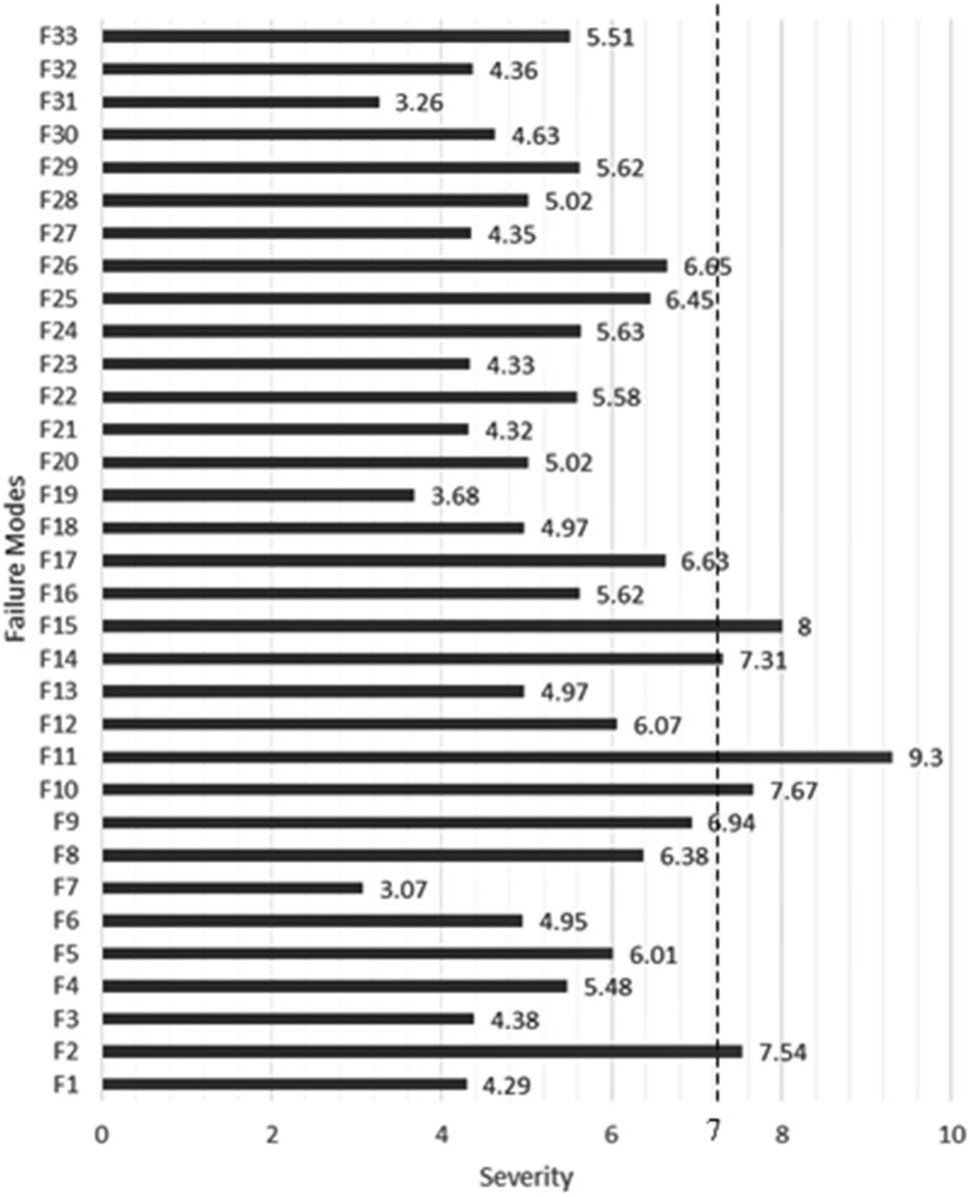
Comparative chart of "Severity" values

The Severity number ranges between 1 and 10. Severity numbers above 7, marked by a dashed line shown in Fig. 4, are highly critical and must be treated regardless of their overall RPN number. According to step 3 of Sect. 2.6 and based on Eq. (7), to obtain the numbers related to the severity of each failure mode, the averages are calculated separately in each of groups A and B and listed in columns *S*_*j*_^*A*^ and *S*_*j*_^*B*^, respectively, in Table 3. Also, the average of these two values is calculated and placed in the third column (*S*_*j*_). Obviously, the average between these two numbers, considering the number of members in each group (20 people in group *A* and 60 people in group *B*), indicates that the influence of each person's opinion in group *A* is more than group *B*.

**Table 3:** Severity number of failure modes

Failure modes	*S*_*j*_^*A*^	*S*_*j*_^*B*^	*S*_*j*_	Failure modes	*S*_*j*_^*A*^	*S*_*j*_^*B*^	*S*_*j*_
F1	3.92	4.65	4.29	F18	4.65	5.29	4.97
F2	8.12	6.95	7.54	F19	3.75	3.61	3.68
F3	4.25	4.51	4.38	F20	4.88	5.16	5.02
F4	5.85	5.11	5.48	F21	4.28	4.35	4.32
F5	6.78	5.24	6.01	F22	5.18	5.98	5.58
F6	4.33	5.56	4.95	F23	4.11	4.55	4.33
F7	3.51	2.63	3.07	F24	4.85	6.41	5.63
F8	6.61	6.14	6.38	F25	6.75	6.14	6.45
F9	7.33	6.55	6.94	F26	6.91	6.39	6.65
F10	7.52	7.81	7.67	F27	4.21	4.49	4.35
F11	9.65	8.95	9.3	F28	4.86	5.18	5.02
F12	5.61	6.52	6.07	F29	5.91	5.33	5.62
F13	5.69	4.25	4.97	F30	4.53	4.72	4.63
F14	7.1	7.51	7.31	F31	2.91	3.61	3.26
F15	8.38	7.61	8	F32	4.51	4.2	4.36
F16	5.32	5.92	5.62	F33	5.11	5.91	5.51
F17	6.75	6.5	6.63				

**Fig. 5:**
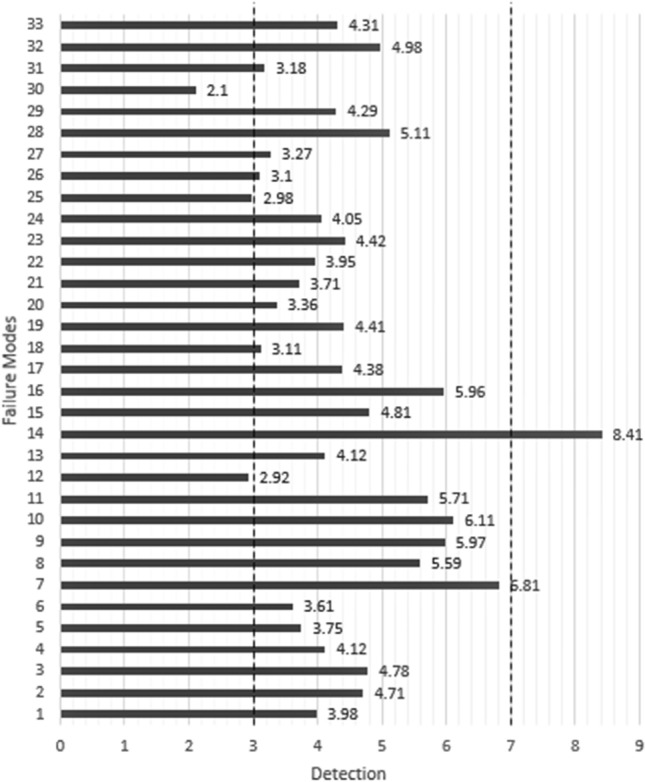
Comparative chart of "Detection" values

Then, for detection number, the average value of the detection numbers assigned to each failure mode by individuals in group *A* is calculated and reported in Table 4. The detection number ranges between 1 and 10. It is divided into three parts: the range 0–3 as easy and obvious diagnosis, the range 3–7 as the average and normal diagnosis, and the range 7–10 as difficult to diagnose. These sections are shown in the diagram with two dashes in Fig. 5.

**Table 4:** Detection numbers of failure modes

Failure modes	*D*_*j*_	Failure modes	*D*_*j*_	Failure modes	*D*_*j*_
F1	3.98	F12	2.92	F23	4.42
F2	4.71	F13	4.12	F24	4.05
F3	4.78	F14	8.41	F25	2.98
F4	4.12	F15	4.81	F26	3.1
F5	3.75	F16	5.96	F27	3.27
F6	3.61	F17	4.38	F28	5.11
F7	6.81	F18	3.11	F29	4.29
F8	5.59	F19	4.41	F30	2.1
F9	5.97	F20	3.36	F31	3.18
F10	6.11	F21	3.71	F32	4.98
F11	5.71	F22	3.95	F33	4.31

According to Sect. 2.6, step 6, the number of repetitions of each failure mode are calculated and presented in Table 5, and the values of *q*_max_ = 61 and *q*_min_ = 9, are determined. Next, using Eq. (9), the center of each occurrence interval is calculated as follows:

$$q^{{\prime }} = \left\{ {11.6, 16.8, 22.0, 27.2, 32.4, 37.6, 42.8, 48.0, 53.2, 58.4} \right\}$$

**Table 5:** Number of repetitions of each failure mode

Failure mode	*q*_*j*_^*A*^	*q*_*j*_^*B*^	*q*_*j*_	Failure mode	*q*_*j*_^*A*^	*q*_*j*_^*B*^	*q*_*j*_	Failure mode	*q*_*j*_^*A*^	*q*_*j*_^*B*^	*q*_*j*_
F1	10	39	49	F12	5	28	33	F23	4	11	15
F2	6	26	32	F13	8	27	35	F24	4	22	26
F3	11	42	53	F14	9	38	47	F25	5	23	28
F4	11	50	61	F15	6	19	25	F26	8	30	38
F5	8	26	34	F16	3	13	16	F27	9	32	41
F6	7	24	31	F17	13	46	59	F28	10	35	45
F7	4	16	20	F18	9	40	49	F29	2	7	9
F8	4	13	17	F19	8	36	44	F30	9	41	50
F9	5	27	32	F20	7	22	29	F31	3	16	19
F10	8	31	39	F21	6	25	31	F32	6	21	27
F11	3	15	18	F22	7	35	42	F33	12	46	58

After it, as mentioned in Sect. 2.6, step 7, we form set $Q = \left\{ {q_{j} \cdot q^{{\prime }} } \right\}$. The occurrence numbers values are obtained for each failure mode, using *k*-means (*Q*,10) in MATLAB R-2013 software into 10 categories. The center of clusters obtained from the *k*-means process is listed in Table 6. The occurrence numbers (*O*) are arranged in Table 7.

**Table 6:** Occurrence numbers for each failure mode

Failure modes	*Q*_*j*_(*O*)	Failure modes	*Q*_*j*_(*O*)	Failure modes	*Q*_*j*_(*O*)
F1	2	F12	6	F23	7
F2	6	F13	10	F24	8
F3	2	F14	2	F25	8
F4	4	F15	1	F26	5
F5	10	F16	7	F27	9
F6	6	F17	4	F28	9
F7	7	F18	2	F29	3
F8	7	F19	9	F30	2
F9	6	F20	8	F31	7
F10	5	F21	6	F32	8
F11	7	F22	9	F33	4

**Table 7:** Occurrence cluster centers obtained from *k*-means process

Cluster no	1	2	3	4	5	6	7	8	9	10
Center	10.3	17.4	23.5	27.4	31.9	34.5	38.2	43.0	49.9	59.3

Then, using Eq. (1), $\left( {{\text{RRPN}}_{j} = S_{j} \times D_{j} \times O_{j} } \right)$, RRPNj values are calculated as shown in Table 8. For modifying the value of RRPN, the MRPN is determined using Eq. (2) by applying the fuzzy inference system “Risk”. This can be seen in Table 9.

**Table 8:** Values of raw risk priority number (RRPN) for failure modes

Failure modes	*S*	*O*	*D*	RRPN	Failure modes	*S*	*O*	*D*	RRPN
F1	4.29	2	3.98	34.15	F18	4.97	2	3.11	30.91
F2	7.54	6	4.71	213.08	F19	3.68	9	4.41	146.06
F3	4.38	2	4.78	41.87	F20	5.02	8	3.36	134.94
F4	5.48	4	4.12	90.31	F21	4.32	6	3.71	96.16
F5	6.01	10	3.75	225.38	F22	5.58	9	3.95	198.37
F6	4.95	6	3.61	107.22	F23	4.33	7	4.42	133.97
F7	3.07	7	6.81	146.35	F24	5.63	8	4.05	182.41
F8	6.38	7	5.59	249.65	F25	6.45	8	2.98	153.77
F9	6.94	6	5.97	248.59	F26	6.65	5	3.1	103.08
F10	7.67	5	6.11	234.32	F27	4.35	9	3.27	128.02
F11	9.3	7	5.71	371.72	F28	5.02	9	5.11	230.87
F12	6.07	6	2.92	106.35	F29	5.62	3	4.29	72.33
F13	4.97	10	4.12	204.76	F30	4.63	2	2.1	19.45
F14	7.31	2	8.41	122.95	F31	3.26	7	3.18	72.57
F15	8	1	4.81	38.48	F32	4.36	8	4.98	173.7
F16	5.62	7	5.96	234.47	F33	5.51	4	4.31	94.99
F17	6.63	4	4.38	116.16

**Table 9:** Values of modified risk priority number (MRPN) for failure modes

Failure modes	*S*	RRPN	MRPN	Failure modes	*S*	RRPN	MRPN
F1	4.29	34.15	14.30	F18	4.97	30.91	59.93
F2	7.54	213.08	63.31	F19	3.68	146.06	50.00
F3	4.38	41.87	14.04	F20	5.02	134.94	45.80
F4	5.48	90.31	16.07	F21	4.32	96.16	13.49
F5	6.01	225.38	50.07	F22	5.58	198.37	37.55
F6	4.95	107.22	26.45	F23	4.33	133.97	45.50
F7	3.07	146.35	19.45	F24	5.63	182.41	16.39
F8	6.38	249.65	52.89	F25	6.45	153.77	50.00
F9	6.94	248.59	57.59	F26	6.65	103.08	42.77
F10	7.67	234.32	64.66	F27	4.35	128.02	50.00
F11	9.3	371.72	86.61	F28	5.02	230.87	53.77
F12	6.07	106.35	27.24	F29	5.62	72.33	40.56
F13	4.97	204.76	49.79	F30	4.63	19.45	42.00
F14	7.31	122.95	56.07	F31	3.26	72.57	50.00
F15	8	38.48	14.30	F32	4.36	173.7	15.14
F16	5.62	234.47	63.31	F33	5.51	94.99	13.43
F17	6.63	116.16	14.04				

At the next step, a total of 41 possible solutions for failure modes were extracted using a survey of people in both groups *A* and *B*. As mentioned in Sect. 2.6, step 10, similar and close values are conceptually unified and arranged in Table 10 as C1 to C41.

**Table 10:** Preventive and corrective actions

Action code	Preventive and corrective action	Action code	Preventive and corrective action
C1	The questions should be designed by the instructor himself	C22	Solving questions does not require unusual tools
C2	The teacher should identify the priorities and objectives of the lesson	C23	Considering internet quality
C3	Coordination between groups in designing questions	C24	Questions and the required format of answers should require minimal storage
C4	All instructors of a course should be engaged in question design	C25	Questions should be straight forward
C5	Exam duration should be considered proportional with the questions	C26	The educator should clarify his assessment policies
C6	The teacher should use the experience of previous years to determine the time of the exam	C27	Introducing the appropriate question and answer reference to the student, to get acquainted with the correct answers
C7	Considering the submission method when planning for its required time	C28	Use question reproduction methods that do not change the nature of the question as well as the overall process of solving it
C8	User friendly examination system	C29	Fine and precise grading steps
C9	Examination system can handle a large amount of participants	C30	Use a set of questions with all three difficulty levels (easy, medium, hard)
C10	Using proper interfaces for the exam	C31	Design questions tailored to the class level
C11	The questions should be conceptual	C32	Provide correct answers to questions for the student after the exam
C12	Identification of online sources available for students during the exam	C33	Before the test, the details should be clearly stated
C13	Using textbooks should be allowed during the exam	C34	Do not be too pessimistic about the student
C14	Using a question bank and assigning questions randomly	C35	Consult with students about the date and time of the exam
C15	Inspecting cheating in answers	C36	Questions should have comparable level to the teaching topics
C16	Authentication by handwrite matching	C37	Mutual understanding between professor and student of each other's situation
C17	Authentication by webcam	C38	The educator should be present at the exam session
C18	Considering general submitting rules	C39	Instead of a final exam, get more quizzes
C19	The grader should not be aware of the identity of the students when grading	C40	Oral test
C20	Fine and precise grading policies	C41	The exam is a combination of oral and written
C21	Selection of a valid scientific reference to correct questions		

After listing the solutions, according to step 11 of Sect. 2.6, in an analogy to the engineering products, the QFD matrix was generated. Failure modes are given as the customer needs, and MRPNs are their priority. Suggested solutions are assumed as technical product characteristics (*C*_*r*_). Then, using average values from groups *A* and *B*, the QFD matrix was completed. The weight (here, priority) and normalized weight of each solution were obtained by applying (13) and (14), respectively. The result is presented in Table 11. As mentioned in step 12, this is the final result of the proposed algorithm. Prioritized actions are listed in Table 12.

**Table 11:** Normalized weight of solutions

Action	*W*_*N*_	Rank	Action	*W*_*N*_	Rank	Action	*W*_*N*_	Rank	Action	*W*_*N*_	Rank
C1	0.0175	31	C12	0.0157	35	C23	0.0342	8	C34	0.0302	11
C2	0.0269	17	C13	0.0124	41	C24	0.0127	40	C35	0.0348	7
C3	0.015	39	C14	0.0198	25	C25	0.0292	12	C36	0.0368	3
C4	0.035	6	C15	0.0326	10	C26	0.0177	30	C37	0.0151	38
C5	0.018	28	C16	0.0367	4	C27	0.0225	23	C38	0.0156	36
C6	0.0231	22	C17	0.0213	24	C28	0.0264	18	C39	0.0178	29
C7	0.0152	37	C18	0.0189	26	C29	0.0273	16	C40	0.028	15
C8	0.0163	34	C19	0.0369	2	C30	0.0283	14	C41	0.0174	32
C9	0.036	5	C20	0.029	13	C31	0.0251	19			
C10	0.0183	27	C21	0.0246	21	C32	0.0251	19			
C11	0.0168	33	C22	0.0327	9	C33	0.037	1

**Table 12:** Normalized change in values of RPN

Failure mode	RRPN	RRPN-normal	MRPN	MRPN-normal	Change
F1	34.15	0.72	14.30	1.10	0.38
F2	213.08	4.48	63.31	4.86	0.38
F3	41.87	0.88	14.04	1.08	0.20
F4	90.31	1.90	16.07	1.23	− 0.66
F5	225.38	4.74	50.07	3.84	− 0.89
F6	107.22	2.25	26.45	2.03	− 0.22
F7	146.35	3.08	19.45	1.49	− 1.58
F8	249.65	5.25	52.89	4.06	− 1.19
F9	248.59	5.23	57.59	4.42	− 0.80
F10	234.32	4.93	64.66	4.96	0.04
F11	371.72	7.81	86.61	6.65	− 1.16
F12	106.35	2.24	27.24	2.09	− 0.14
F13	204.76	4.30	49.79	3.82	− 0.48
F14	122.95	2.58	56.07	4.31	1.72
F15	38.48	0.81	59.93	4.60	3.79
F16	234.47	4.93	50.00	3.84	− 1.09
F17	116.16	2.44	45.80	3.52	1.08
F18	30.91	0.65	13.49	1.04	0.39
F19	146.06	3.07	37.55	2.88	− 0.19
F20	134.94	2.84	45.50	3.49	0.66
F21	96.16	2.02	16.39	1.26	− 0.76
F22	198.37	4.17	50.00	3.84	− 0.33
F23	133.97	2.82	42.77	3.28	0.47
F24	182.41	3.83	50.00	3.84	0.01
F25	153.77	3.23	53.77	4.13	0.90
F26	103.08	2.17	40.56	3.11	0.95
F27	128.02	2.69	42.00	3.23	0.53
F28	230.87	4.85	50.00	3.84	− 1.01
F29	72.33	1.52	15.14	1.16	− 0.36
F30	19.45	0.41	13.43	1.03	0.62
F31	72.57	1.53	15.79	1.21	− 0.31
F32	173.70	3.65	45.32	3.48	− 0.17
F33	94.99	2.00	16.33	1.25	− 0.74

## Discussion

According to Table 1, if the severity numbers are in the range of 7–10, they express the major effect of the failure mode on the end-user. Therefore, the number 7 is marked in the diagram with a dividing line as the "threshold". The highest severity numbers in the critical region (F11, F15, F10, F2, and F14) show that the most confusing and dissatisfying effect in an online test is related to *credibility and fraud prevention*.

Also, if the scoring is not entirely consistent with a specific policy, it can cause severe adverse effects. On the other hand, designing test questions by someone other than the instructor can cause serious problems.

According to Tables 1 and 4 and Fig. 5, only one failure mode is within the difficult detection range, which is F14 (*inconsistency in grading*). It is quite logical that if the question designer (who should be the instructor himself/herself) does not provide a specific key to grading the exam answer scripts, it will not be easy to identify the consistency of the results.

**Fig. 6:**
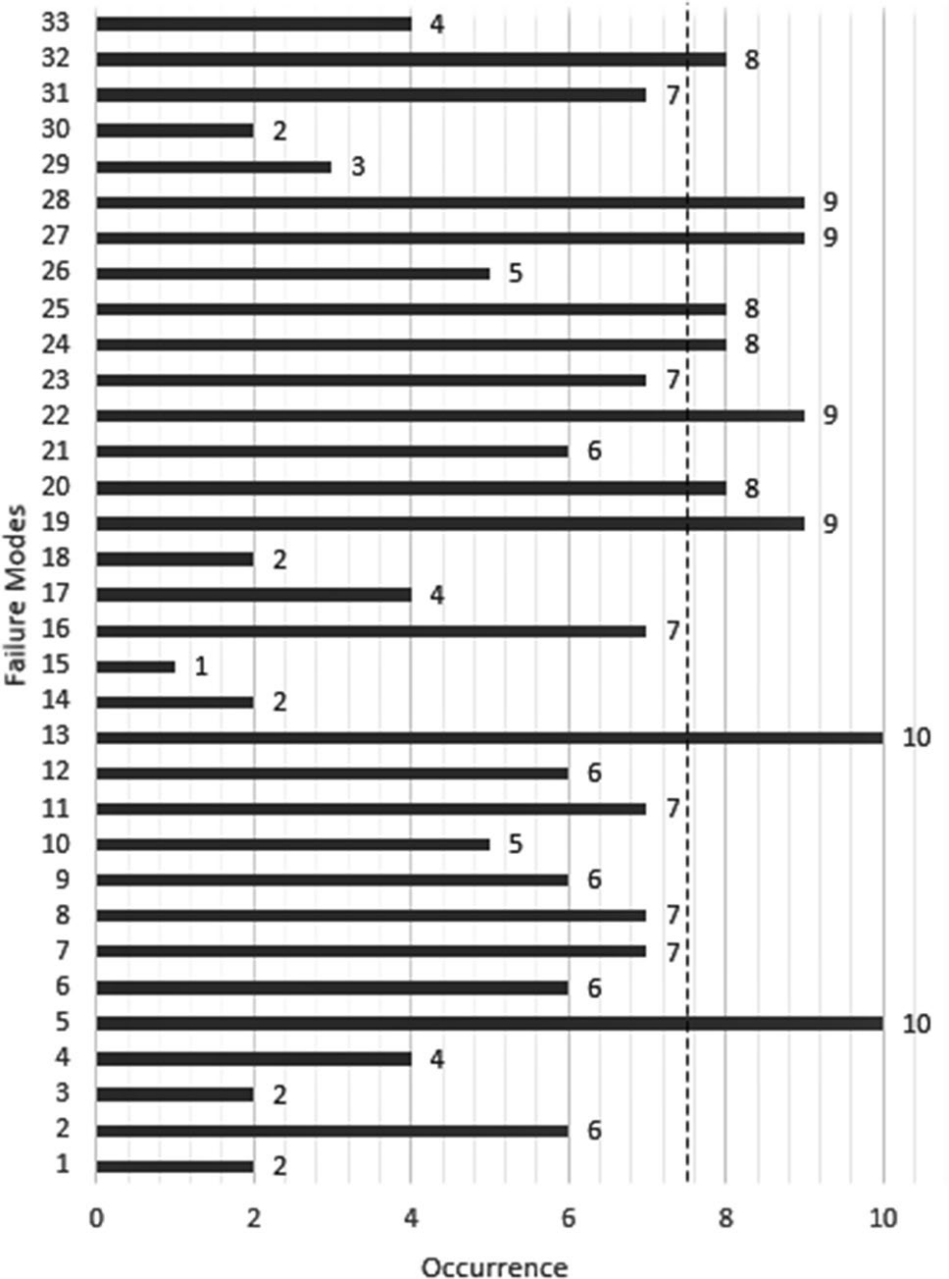
Comparative chart of "Occurrence" values

Considering the numbers in Table 6, which is derived from the proposed *k*-means system for determining occurrence numbers, and Fig. 6, which is a comparative graph of occurrence values, the failures with the most likely to occur (Containing F13, F5, F19, F22, F27, and F28) do not have high severity. Therefore, it can be concluded that the online exams that have been held so far are mainly at an acceptable level of customer satisfaction, and efforts should be more focused on improving the current level.

Also, the presence of these failure modes in the list of the high probability shows that the main reasons for the occurrence of failure modes are the way the instructor teaches, the exact expression of expectations, and the appropriateness of time with the questions.

**Fig. 7:**
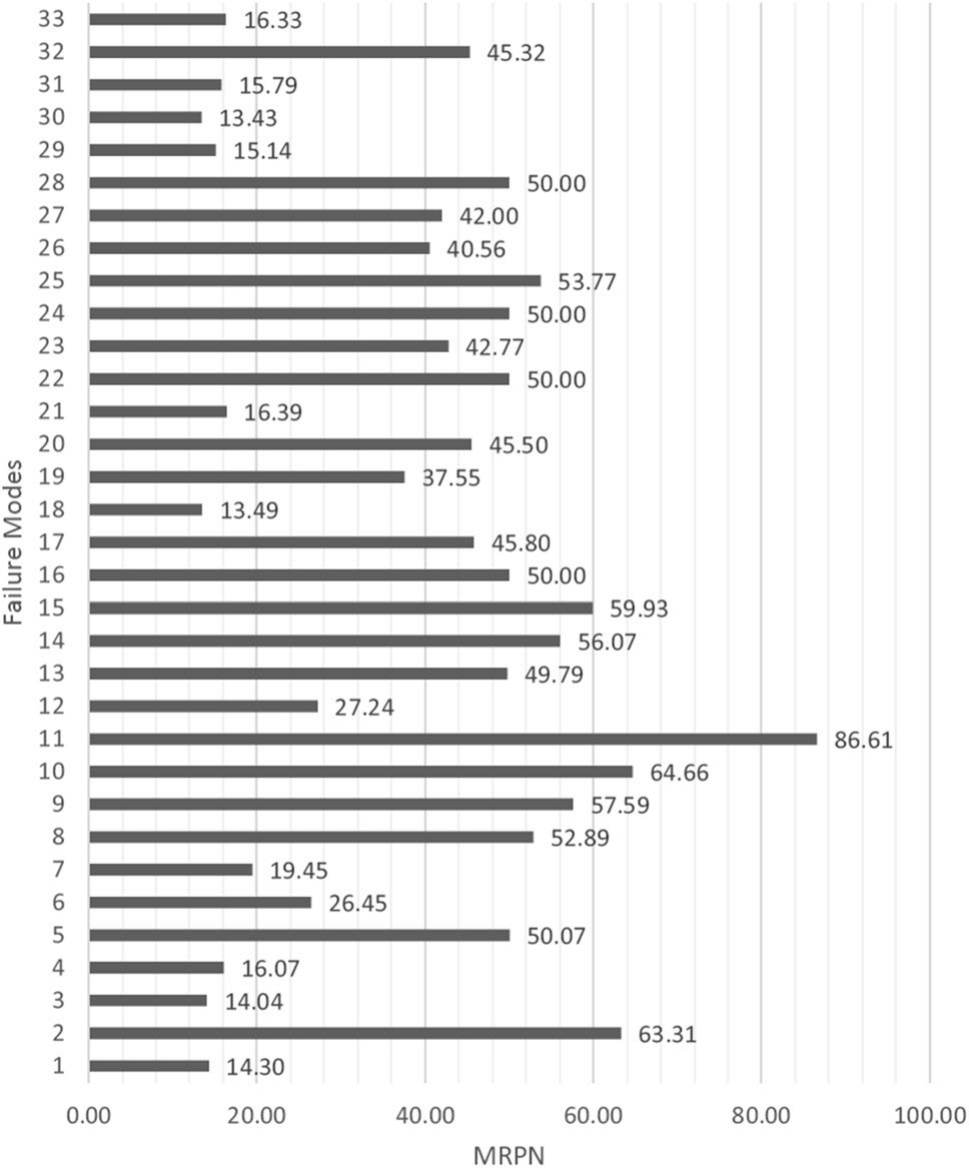
Comparative chart of "MRPN" values

According to Table 9 and Fig. 7, failure modes with the highest MRPN (modified values of the risk priority number) containing F11, F10, F2, F15, and F9, the main critical issue related to an online exam is *cheating*, which can undermine the validity and the fairness of an exam. Also, the presence of heterogeneity or an incorrect key can disrupt the whole result. On the other hand, if the questions are not from the taught topics, the test is invalid. In general, it can be said that if cheating is prevented, we can hopefully accept the appropriateness of the online exam.

**Fig. 8:**
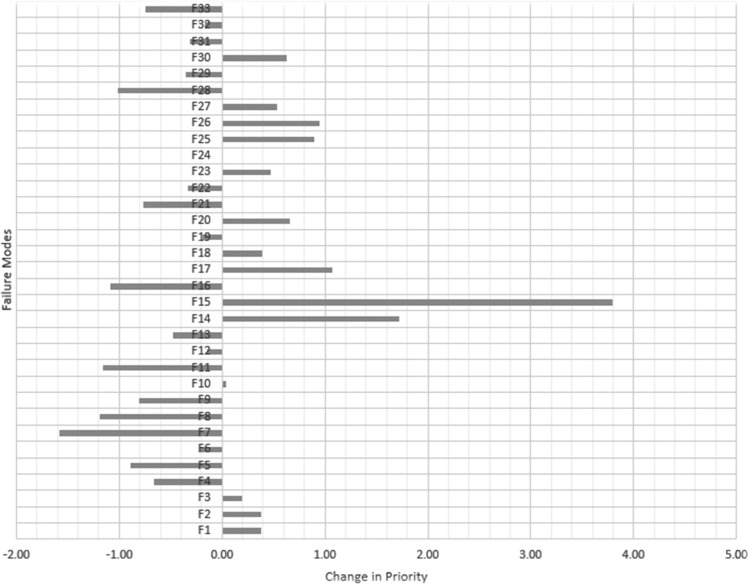
Comparative chart of "RPN" change (Influence of RPN Modification)

To evaluate the effectiveness of the fuzzy inference system in making more appropriate criteria for comparing the criticality of each failure mode, we should study the cases with the most changes in the initial RPN number. To do this, both RRPN and MRPN values should be normalized. The normalization range here is 1–100, depending on the numbers available. From Table 12 and Fig. 8, the most changes in the order of increasing priority occurred in F15, F14, and F17. These failure modes do not have very large RRPNs, but their severity value is high. Therefore, the fuzzy inference system has led them to increase priority. This indicates the correct operation of the modifier system.

Time and cost constraints prevent us from implementing all corrective and preventive actions (C1 to C41 in Table 10). Therefore, we need to prioritize them. High-priority solutions will be actions that can prevent more hazardous failure modes. Table 11 shows that the most important actions to maximize customer (including faculty, assistants, and students) satisfaction during an online test (containing C33, C19, C36, C16, and C9), the exact expressing of the expectations in the test and evaluation methods, holding the exam with sufficient supervision at the right time and place, designing exam questions and key by the instructor him/herself and also, the existence of appropriate infrastructure, can prevent potential problems in an online test.

It is also emphasized that, since this method is based on FMEA, the provided solutions have a preventive aspect, leading to a reduction in adverse effects in emergencies such as the recent pandemic of the COVID-19.

## Limitations and future scope of the work

Because in this study, all surveys are based on crisp numbers, there may be some deviation in the conclusions. Subsequent studies using fuzzy logic (which is closer to the human mental structure in terms of ambiguity and psycholinguistics) could yield better results.

In the implementation of the second part of the algorithm, the surveys were conducted only for the students of Sharif University of Technology (which is an engineering university). Further studies at various universities, including all four departments of Mathematical and Technical Sciences, Medical Sciences, Humanities and Arts, will have a significant impact on the comprehensiveness of the results.

Using different methods of data mining and data processing, such as AHP, ANP, and DEMATEL, can be very helpful in better analyzing the results.

## Conclusion

The COVID-19 pandemic and the need to adhere to health protocols, including avoiding crowded gatherings, have led to a sudden and growing demand for online college classes. The assessment process is one of the most important components of any academic course, especially when a crisis exists. Because of Time and cost constraints, implementing all proposed solutions is impossible and makes it necessary to prioritize them.

In this study, a fair online exam is defined as a test that leads to customer satisfaction (including faculty, assistants, educational system, and students). Then, in analogy to an engineering product, the product design process is performed on it. As the first stage, the FMEA process, which is a preventive method in identifying potential failure modes, is employed to find the potential failure modes, their severity, occurrence, and preventive detection method. Then, the risk priority number of each case is calculated. The K-means method, which is an unsupervised clustering algorithm, has been used to eliminate or minimize the effects of conflicting conditions in assigning occurrence-related numbers. The results show the effectiveness of these two modifications on determining the risk priority of failure modes. Therefore, the QFD algorithm was used to determine the weight of each solution and prioritize its application by considering the proposed solutions as technical characteristics of an engineering product.

The results show that if the taught topics and exam titles are consistent, the instructor's expectations of the students are clear, there is a clear assessment policy, the test is held under adequate supervision at the right time and place, and with the appropriate infrastructure, the test questions are designed by the instructor him/herself, the maximum satisfaction of the stakeholders will be obtained. According to the provided definition, it will lead to an increase in the validity of the online test.
